# Study of Variation of *ACOX1* Gene Among Different Horse Breeds Maintained in Iran

**DOI:** 10.3390/ani14243566

**Published:** 2024-12-10

**Authors:** Shayan Boozarjomehri Amnieh, Ali Hassanpour, Sina Moghaddam, Fatemeh Sakhaee, Katarzyna Ropka-Molik

**Affiliations:** 1 Faculty of Veterinary Medicine, Tabriz Medical Sciences, Islamic Azad University, Tabriz 5159115705, Iran; shayanbzj@gmail.com; 2 Department of Clinical Science, Tabriz Medical Sciences, Islamic Azad University, Tabriz 5159115705, Iran; 3 Department of Internal Medicine, Faculty of Veterinary Medicine, University of Tehran, Tehran 1417935840, Iran; smoghaddam33@gmail.com; 4 Department of Mycobacteriology and Pulmonary Research, Pasteur Institute of Iran, Tehran 1963737611, Iran; 5 Department of Animal Molecular Biology, National Research Institute of Animal Production, Krakowska 1, 32-083 Balice, Poland; katarzyna.ropka@iz.edu.pl

**Keywords:** Arabian horses, Thoroughbred, KWPN, Caspian, Kurdish, Turkmen, genetic marker, peroxisomal acyl-coenzyme a oxidase 1

## Abstract

The *ACOX1* gene is vital for fatty acid metabolism and is linked to environmental stress and physical exertion adaptation. The p.Asp237Ser variant (rs782885985) in *ACOX1* is associated with increased enzyme activity and reactive oxygen species (ROS) levels. This study examined the *ACOX1* polymorphism across six horse breeds in Iran: Arabian, Thoroughbred, KWPN, Caspian, Kurdish, and Turkmen. The goal was to identify differences in *ACOX1* genotype distribution, potentially serving as genetic markers under selection pressure related to breed-specific traits. In a sample of 324 horses, genomic DNA was analyzed using PCR-RFLP, revealing three genotypes (TT, TG, GG). The GG genotype was most common in Kurdish and Arabian horses (86% and 70%, respectively), while the TT genotype was prevalent in Turkmen (24%) and Thoroughbred horses (23%). The T allele’s frequency in Thoroughbred and Turkmen horses suggests that ACOX1 may be under selection pressure for phenotypic traits. Differences in genotype distribution were confirmed among breeds, with no sex-based association. The study concludes that *ACOX1* is a potential genetic marker for horse performance and adaptability, emphasizing the importance of genetic diversity in breeding programs.

## Simple Summary

Horses have played a vital role in human history, evolving through domestication and selective breeding to meet various utility needs, from transportation and labor to leisure and competitive sports. Over time, selective pressures shaped the genetic basis of equine traits, resulting in more than 400 distinct breeds with unique adaptations to environmental conditions and physical exertion. Identifying genetic markers under selection is crucial for understanding these adaptations and improving breed-specific performance. This study examines the variation of the *ACOX1* gene across different horse breeds in Iran and its connection to breed-specific traits. By exploring the genetic polymorphisms of *ACOX1*, we can gain insights into how different breeds may adapt metabolically to their environment and perform physical exertion tasks differently.

## 1. Introduction

The relationship between humans and horses has a rich and diverse history. Horse domestication and breeding are closely related to their utility type and direction of use. Horses play an essential role in the development of civilization as a source of workforce, transportation, and communication. Horses have also been critical in military history, from cavalry to logistics and transport [1]. For centuries, horses have been increasingly valued as companions, of which importance now comes to the fore [2]. Horses quickly acquired a multifaceted role, unlike many other domesticated ungulates, which are primarily bred for purposes such as meat, milk, or wool production. Nowadays, horses still serve as a food source or are utilized as working companions in rural settings. However, they are primarily used in leisure activities, equestrian sports, and shows [3]. In recent years, therapeutic riding programs have gained considerable popularity, highlighting yet another beneficial use of horses and horseback riding [4].

On the one hand, numerous studies over the past decade have explored the genetic basis of various equine adaptations to physical exertion. During domestication and breeding, horses were subjected to artificial selection pressure to develop traits desirable for specific uses [5]. These adaptations largely stem from selective pressures that have shaped horses’ natural abilities in response to human needs and environmental factors. This process has significantly altered phenotypes and genetic diversity, leading to the development of over 400 distinct horse breeds [6].

Given the influence of genetic diversity and the environmental bottleneck effect, it is crucial to identify genetic markers that are under selection pressure and associated with specific phenotypes in different horse breeds. This approach is justified to better understand the genetic basis of breed-specific traits. This study examined six different horse breeds, each representing various utility types, including Arabian, Thoroughbred, Koninklijk Warmblood Paard Nederland (KWPN), Caspian, Kurdish, and Turkmen horses maintained in Tabriz, Marand, Anzali, Karaj, Tehran and Gonbad-e Kavoos regions.

Arabian horses, believed to be among the oldest and most influential breeds globally, have a rich history. While their origins remain unclear, the Arabian Peninsula, notably Yemen, Saudi Arabia, and Iran, is often cited as their birthplace. However, other regions of the Middle East are also mentioned [7]. Modern Arabian horses possess distinctive physical traits, including a concave facial profile, wide-set eyes, arched neck, and high tail carriage [8]. Historical photographs suggest that selective breeding has gradually enhanced these traits, particularly in non-ridden horses, such as those used in Arabian horse beauty competitions. Known for their heat tolerance and endurance, Arabian horses excel in long-distance races, with some covering distances of up to 160 km in around eight hours.

Dutch Warmblood horses’ ancestry is traced back to the Groninger and Gelderlander breeds, which were common in the Netherlands before World War II. The modern KWPN studbook, though relatively young compared to others, has been at the forefront of specialized breeding. Most Warmblood registries focus on improving performance in dressage and show jumping, with the KWPN evaluating genetic potential based on competition scores [9].

Thoroughbreds, prized as racehorses for centuries, have undergone intense selection for athletic phenotypes conducive to superior performance on the racetrack. While environmental factors play a significant role, genetic factors influence athletic capabilities. Breeding strategies are based on the assumption that racing ability is heritable, with numerous studies demonstrating how Thoroughbreds have developed specific physiological features that enhance athleticism and performance during exercise [10,11,12,13,14].

The Caspian horse typically measures around 120 cm at the withers, featuring a concave profile, vaulted forehead, straight and short back, and a high-set croup and tail. The Caspian breed, originating from the mountainous regions of Northern Iran, is renowned for its resilience, athleticism, and agility. Known for their durable hooves, Caspian horses rarely require shoeing unless exposed to particularly challenging or rocky terrains. The Caspian breed is considered unique among equine breeds as it does not easily fit into conventional ancestral classifications, making it a valuable genetic link to ancient horse lineages. Consequently, the Caspian is regarded as one of the rarest horse breeds.

The Turkmen horse, native to the Central Asian steppes, has significantly influenced the development of many modern horse breeds, including the Thoroughbred. Variants such as the Akhal-Teke, Iomud, Goklan, and Nokhorli are direct descendants of the Turkmen horse [15]. These horses, characterized by a slender build, straight profile, elongated neck, and strong legs, have notably contributed to the development of the Thoroughbred, mainly through the Byerley Turk bloodline. Turkmen horses were brought to England by soldiers stationed in the East, who recognized their endurance and disease resistance, which made them ideal for racing purposes. Additionally, previous publications have suggested that both Caspian and Turkmen horses may carry genetic markers that can be considered ancestral to other oriental horse breeds [15,16,17].

The Kurdish horse, along with other breeds such as the Caspian, Turkmen, Assil (also referred to as Persian Arab), and Dare Shuri, represents one of Iran’s principal indigenous horse breeds. Originating from the rugged terrains of Western Iran, the Kurdish horse has evolved over millennia to thrive in harsh environmental conditions. Local breeders have refined the breed through selective breeding, emphasizing adaptability to mountainous environments and rhythmic movement [18].

The *ACOX1* gene, encoding a crucial enzyme involved in fatty acid metabolism, holds significance for organisms enduring prolonged starvation and harsh conditions. Variations within *ACOX1,* revealed through RNA sequencing, may reflect adaptation to environmental stressors driven by selection pressures. Recent studies have delved into the genetic underpinnings of equine adaptations to exertion, revealing targets of domestication and breed differentiation at the genomic level. Genomic regions associated with skeletal and cardiac muscle, energy metabolism, and lipid deposition underscore the multifaceted nature of horse genetics [19,20]. The *ACOX1* gene, located on chromosome 17q25.1, encodes peroxisomal acyl-coenzyme A oxidase 1. A previous study utilized a single-nucleotide polymorphism (SNP) located in the *ACOX1* gene as a potential genetic marker related to metabolism, thermogenesis, and exercise phenotype [21]. The single nucleotide polymorphism rs782885985 within the *ACOX1* gene is a missense variant that results in an amino acid substitution. Specifically, this SNP leads to the change in an aspartic acid (Asp) to serine (Ser) at position 237 of the protein (p. Asp237Ser).

The present report focuses on examining *ACOX1* gene variation across six horse breeds—Arabian, Turkmen, KWPN, Thoroughbred, Kurdish, and Caspian—each subject to distinct selection pressures to evaluate the potential association of ACOX1 with horse performance. According to previous reports, we hypothesized that variations within the *ACOX1* gene might elucidate connections to breed-specific traits influenced by metabolism, energy demands, and environmental factors.

## 2. Materials and Methods

### 2.1. Sample Collection and DNA Extraction

**Figure 1:**
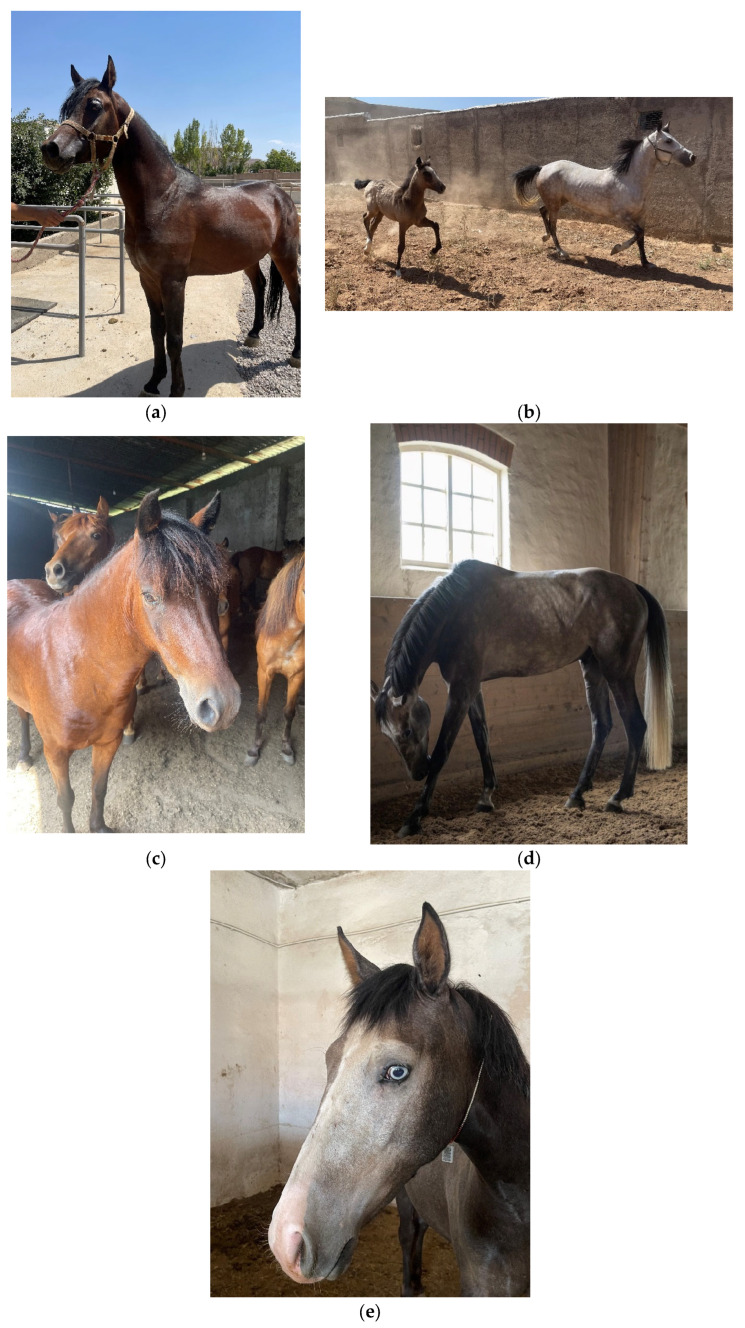
(**a**) Arabian horse in Tabriz, Iran. This photograph was taken by the author Shayan Boozarjomehri Amnieh. (**b**) Kurdish horse in Marand, Iran. This photograph was taken by the author Sina Moghaddam. (**c**) Caspian horse in Anzali, Iran. This photograph was taken by the author Sina Moghaddam. (**d**) KWPN horse in Tehran, Iran. This photograph was taken by the author Shayan Boozarjomehri Amnieh. (**e**) Turkmen horse in Gonbad-e-Kavoos, Iran. This photograph was taken by the author Shayan Boozarjomehri Amnieh.

Between June 2023 and September 2023, a total of 324 horses were included in the study from six cities of IRAN, namely Tehran, Anzali, Karaj, Marand, Tabriz, and Gonbad-e-Kavoos. There were Arabian, *n* = 90; Kurdish, *n* = 73; Caspian, *n* = 21; KWPN, *n* = 50; Thoroughbred, *n* = 35; and Turkmen, *n* = 55 (Figure 1) horses. Whole blood from each horse was sampled and prepared in a sterilized EDTA test tube with a volume of approximately 5 mL. According to the protocol, genomic DNA was isolated from whole blood using a DNA kit (SinaCloneBioScience, Tehran, Iran). DNA quantity and quality were checked using a NanoDrop 2000 spectrophotometer (ThermoFisher Scientific, Waltham, MA, USA). The DNA was stored at −20 °C until further analysis.

This study’s protocol was developed according to the ethical principles approved by the international committees for the protection of animal welfare (Code: IR.IAU.TABRIZ.REC.1402.273).

### 2.2. Identification of ACOX1 Polymorphism and Genotyping

The PCR restriction fragment length polymorphism (PCR-RFLP) method was developed to analyze the selected polymorphism -rs782885985- in exon 2 of the *ACOX1* locus. Specific endonucleases were used to recognize particular gene variants. The PCR reaction for the *ACOX1* gene was performed in a total volume of 20 microliters (μL). This reaction mixture included 10 μL of Master Mix (Ampliqon, Odense, Denmark), 0.5 μL of each primer (Forward: CAGCTGTGATTACGGGAGGT and Reverse: TGAAAACGTGCAGTTTGAGC, 10 pmol), which was used to amplify the *ACOX1* gene region [19]. Additionally, 1.2 μL of Enhancer (Pishgam Biotech, Tehran, Iran), 3 μL of DNA, and 4.8 μL of deionized sterile water (DW) were included in the reaction.

**Figure 2:**
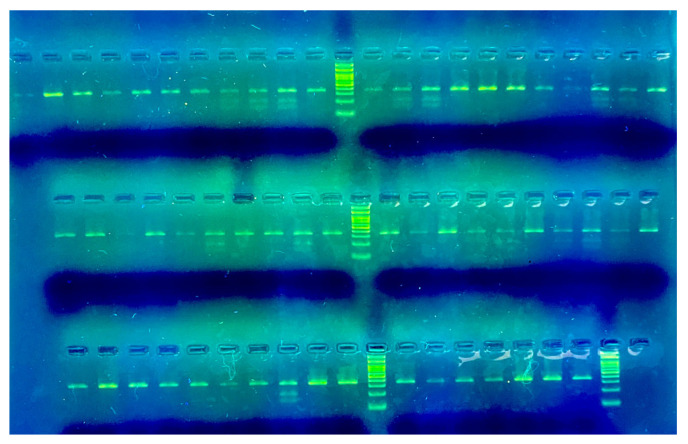
The banding pattern observed on the 3% agarose gel after digestion with DdeI endonuclease confirms the genotypes as follows: GG genotype: A single band is visible at 342 bp, representing the undigested fragment. TG genotype: Three bands are visible at 342 bp, 197 bp, and 145 bp, indicating the presence of both alleles. TT genotype: Two bands are visible at 197 bp and 145 bp, as the 342 bp fragment is absent due to complete digestion.

PCR was performed on a Sensoquest lab-cycler (www.sensoquest.de) as follows: 98 °C for 10 min, 98 °C for 15 s, 57 °C for 30 s, 35 cycles of 72 °C for 30 s, and a final extension at 72 °C for 5 min. Then, PCR products were digested overnight at 37 °C with DdeI endonuclease and separated in 3% agarose gel to obtain the profiles of specific bands for both alleles: T–197, 145 bp; G–342 bp (Figure 2).

This pattern differentiates the genotypes based on the specific cleavage of the PCR product by DdeI.

The presence of polymorphism in the *ACOX1* locus was confirmed using Sanger sequencing, and the significance of differences in genotype distributions (MEGA-7) among the breeds of horses included in this study was calculated using the chi-square test (SPSS-26).

## 3. Results

### 3.1. Genotype Distribution

The horses included in this study exhibited three genotypes (GG, TT, TG). Out of 324 horses, 40 had the TT genotype (12.34%), 79 had the TG genotype (24.38%), and 205 had the GG genotype (63.27%) (Table 1). The GG genotype was predominantly present in Kurdish and Arabian horse populations, with frequencies of 86% and 70%, respectively. KWPN and Caspian horses followed with GG genotype frequencies of 62% and 52%, exhibiting the same allele frequencies (T = 0.29, G = 0.71). Notably, the Arabian breed showed a high GG genotype frequency (70%), while the Thoroughbred breed demonstrated a more balanced distribution of genotypes, albeit deviating from Hardy–Weinberg equilibrium (HWE).

The TT genotype, representing the wild-type allele, was most frequent in the Turkmen breed (24%), followed by Thoroughbred (23%) and KWPN horses (20%). In other breeds, the TT genotype was considerably less common. Kurdish horses showed the highest frequency of the GG genotype (86%) with the lowest frequency of the TT genotype, with TG heterozygotes making up only 12% of the population. Their population appeared to maintain equilibrium according to HWE. By contrast, Thoroughbred, Turkmen, Kurdish, and Arabian horses exhibited similar genotype distributions, with Kurdish horses being the most genetically homogeneous, showing a high degree of monomorphism.

The TG genotype was observed at the highest rate in Caspian and Turkmen horses, both with a frequency of 38%. The analysis of genotype distribution and allele frequencies across different horse breeds revealed several significant genetic variations and deviations from Hardy–Weinberg equilibrium. The populations of Arabian, Thoroughbred, and KWPN horses did not conform to Hardy–Weinberg expectations (Table 1).

**Figure 3:**
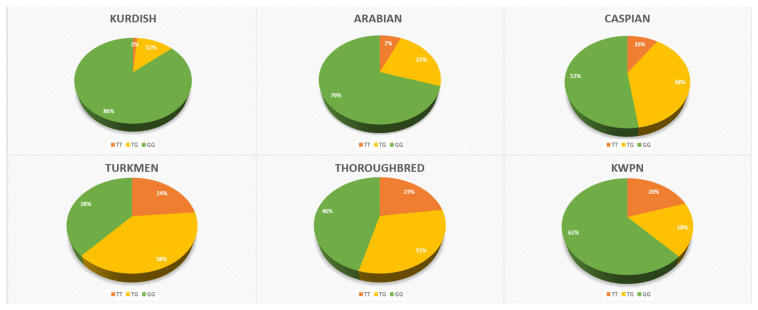
Distribution of ACOX1 genotypes by breed.

The chi-square test confirmed significant differences in genotype distribution between several breeds (Table 2 and Table 3). Notably, Turkmen horses show significant differences from all other breeds but Thoroughbreds. Similarly, Kurdish horses differ significantly from KWPN, Caspian, and Arabian breeds (Figure 3 and Table 3).

### 3.2. The Chi-Square Tests for Independence

For most breeds (Arabian, Caspian, KWPN, Kurdish, and Thoroughbred), the *p*-values are well above the threshold of 0.05. This suggests no statistically significant association exists between allele frequency (GG, TG, TT) and sex (mare, stallion) in these breeds. The distribution of alleles appears to be independent of sex within these breeds.

**Figure 4:**
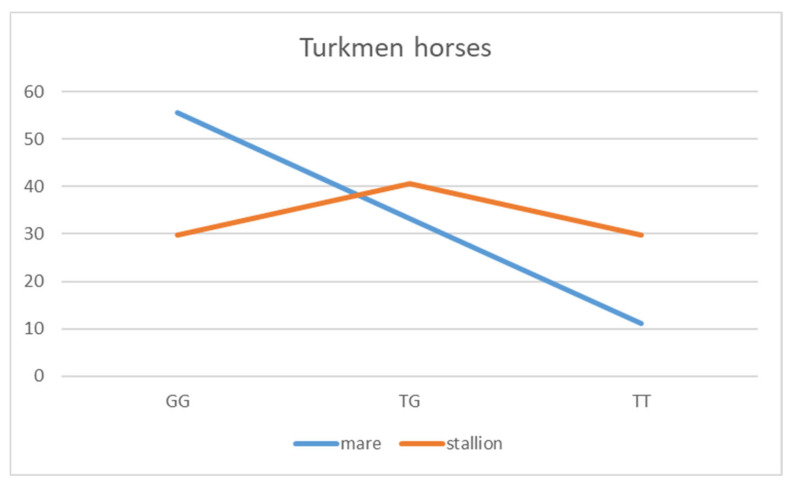
Distribution of genotypes for mares vs. stallions in Turkmen horses. The *Y*-axis represents percentages related to genotype.

Specifically, we tested for an association between sex and allele frequency. The frequency of sex differences in the Turkmen breed showed a *p*-value of 0.13, which, although not below the threshold of 0.05, is lower than the other breeds. This suggests a potential trend towards an association between allele frequency and sex that may warrant further investigation (Figure 4).

## 4. Discussion

The Peroxisomal acyl-CoA oxidase (*ACOX*; EC 1.3.3.6) serves as the initial enzyme in the fatty acid beta-oxidation pathway, catalyzing the conversion of acyl-CoA to 2-trans-enoyl-CoAs [22]. Fatty acid breakdown predominantly occurs through the beta-oxidation cycle in most living organisms. In mammals, beta-oxidation occurs in both mitochondria and peroxisomes [23]. In the liver, the peroxisomal acyl-CoA oxidase is responsible for the classical beta-oxidation pathway under the transcriptional regulation of peroxisome proliferator-activated receptor alpha (PPAR alpha). Studies on mice lacking PPAR alpha, peroxisomal fatty acyl-CoA oxidase, and certain enzymes in the two peroxisomal beta-oxidation pathways highlight the crucial roles of PPAR alpha and classical peroxisomal fatty acyl-CoA oxidase in energy metabolism, as well as in the inception of some diseases such as hepatic steatosis, or liver cancer [24].

Moreover, PPAR alpha targeted genes are the main factors controlling lipid metabolism and maintaining energy homeostasis, which translates into general metabolism and exercise predisposition. The *ACOX1* protein is pivotal as a rate-limiting enzyme in the peroxisomal fatty acid beta-oxidation pathway. Highlighting the importance of the *ACOX1* gene, it is particularly relevant in the context of peroxisomal neurodegenerative disorders. For example, pseudo neonatal adrenoleukodystrophy (P-NALD) is characterized by a deficiency in acyl-coenzyme A oxidase 1 (*ACOX1*). This deficiency results in the accumulation of very long-chain fatty acids (VLCFA) and leads to inflammatory demyelination. *ACOX1* is vital for the α- and β-dehydrogenation of a variety of acyl-CoA esters, including those from dicarboxylic acids, eicosanoid derivatives, and saturated VLCFA [25]. Moreover, equine atypical myopathy (AM) is induced by hypoglycin A intoxication. In turn, hypoglycin A is metabolized into methylene cyclopropyl acetic acid-CoA (MCPA-CoA), a potent inhibitor of acyl-CoA dehydrogenase. Moreover, abnormalities in fatty acid metabolism have been identified in numerous cases of exertional rhabdomyolysis [26].

Missense variants like rs782885985 can significantly affect protein function. In the case of *ACOX1*, the p. Asp237Ser variant has been associated with increased enzyme activity and elevated levels of reactive oxygen species (ROS), contributing to axonal loss and neurodegeneration, as seen in conditions like Mitchell syndrome (MITCH).

**Figure 5:**
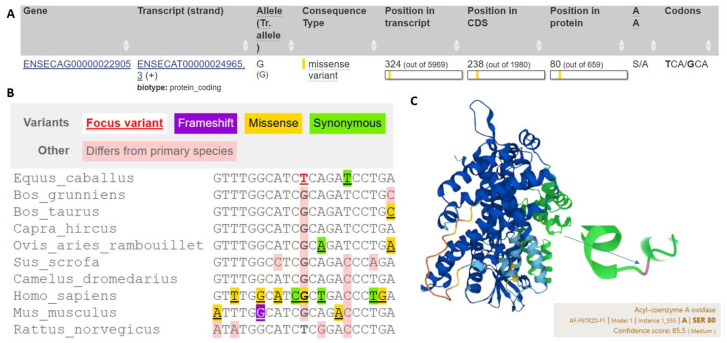
Localization of SNP- Acyl-coenzyme A oxidase, N-terminal domain. The phylogenetic analysis of ENSECAP00000020757.3:p.Ser80Ala (rs782885985) polymorphisms across different species: the exact localization of mutation site in the gene, transcript ((**A**), Ensembl database) and protein chain ((**C**), UniProtKB database); F6TRZ0 Equus Caballus reference); the conservation analysis of rs782885985 SNP across species ((**B**), Ensembl database).

Figure 5 illustrates a missense variant in the equine genome, specifically within a gene encoding the enzyme acyl-coenzyme A oxidase, which plays a critical role in lipid metabolism. Part A of the figure provides detailed information about the variant, including gene and transcript IDs, allele composition, and the specific nucleotide change. This variant results in a codon alteration from “TCA” to “GCA”, causing a substitution of serine with alanine at amino acid position 80. Such a substitution could potentially impact the structure or function of the protein, depending on the structural role of serine in this position.

Part B presents a multi-species alignment to highlight the conservation of the sequence across various species, including *Equus caballus* (horse), *Bos grunniens* (yak), and *Homo sapiens* (human). Different colors indicate variant types, such as missense (yellow) and frameshift (purple), demonstrating evolutionary divergence or conservation in this region. This comparative analysis suggests that the equine variant may have functional significance, as indicated by the conservation of specific nucleotides in closely related species.

Part C shows a 3D model of the acyl-coenzyme A oxidase protein with the variant site marked, providing structural context to the amino acid substitution. The confidence score of 85.5 indicates moderate reliability in the structural model. The serine-to-alanine substitution at position 80 is highlighted within the structure, suggesting possible effects on the enzyme’s stability or interaction with substrates. This structural insight, combined with evolutionary analysis, underscores the potential functional implications of this variant in horses and contributes to understanding the metabolic adaptations or susceptibilities in equine species.

Due to its function, the *ACOX1* gene is considered a marker in horses related to performance ability [19,27]. In challenging environmental conditions such as low temperatures and limited food resources, feral populations of herbivores can lower their metabolic rate and energy requirements [28]. Different breeds of horses, kept under varying conditions and subjected to diverse selection pressures, may exhibit variations in the ACOX1 gene locus associated with their predispositions.

The results showed that several breeds, including Arabian, KWPN, Thoroughbred, and the overall population, present significant deviations from Hardy–Weinberg equilibrium. This suggests that the observed distribution of genotypes for the ACOX1 gene may be shaped by factors such as selection, genetic drift, or non-random mating. Conversely, breeds like Caspian and Kurdish exhibit Hardy–Weinberg equilibrium, indicating a stable allele frequency within these populations. The similarity in allele frequencies between KWPN and Arabian horses could indicate shared genetic influences. This may arise from either a common ancestral lineage or similar selective pressures driven by their utility. Both breeds, despite their distinct histories, have been selectively bred for performance traits like endurance, agility, and elegance, which are traits crucial for both dressage (in KWPN) and endurance (in Arabians). The overlap in these selective traits may lead to similar allele frequencies, reflecting convergent breeding goals rather than direct relatedness.

Previous examination of the g.6105340T>G polymorphism in various horse breeds revealed a higher prevalence of the G allele in breeds adapted to specific environmental conditions [19]. Similarly, in the Iranian horse breeds we investigated—Caspian, Kurdish, and Arabian horses, known for thriving in challenging environments like deserts—there is evidence of enhanced fatty acid metabolism due to natural selection. Another report indicated an association of the *ACOX1* T allele with flat racing performance in Arabian horses [27]. The frequency of the TT genotype in the previously investigated Arabian population was 1.7% [19]. The 7% prevalence of the TT horses observed in the Arabian horse population in this study suggests that this breed has not been subjected to strong selection pressure for phenotypic traits associated with the T allele. In contrast, the Thoroughbred breed has experienced significant selection pressure for traits enhancing racing ability, reflected in the high frequency of the T allele. These findings support previous research indicating that the T variant influences flat racing performance [27]. The results are consistent with a previous study that reported a frequency of 42.5% for the TT genotype in Thoroughbred horses [27]. In the present study, Turkmen horses, known for their exceptional racing ability, exhibited the highest frequency of the TT genotype at 24%. These data collectively suggest a strong association between the T allele and traits associated with speed and racing performance in these horse breeds. The prevalence of the T allele in Thoroughbred and Turkmen horses demonstrated that *ACOX1*, together with other genes involved in PPAR signaling pathways, might be under selection pressure due to the energy metabolism, which is non-negligible for flat racing.

The *ACOX1* gene is under positive selection in Thoroughbred horses due to its localization within significant genome regions under selection and localized on chromosome 11 [29]. *ACOX1* variations could be considered a genetic marker related to gallop-racing performance traits, indicating the need for further research [27]. Our results indicated that such an association study should be performed on Turkmen horses to verify the usefulness of the investigated marker. Though a small and ancient breed, the Caspian horse shares notable genetic links with Thoroughbreds and Turkmen horses. This connection aligns with the historical context of horse breeding and migration across regions like Persia (modern-day Iran), where these breeds may have shared common ancestors. The genetic contributions of the Caspian horse to early horse populations, particularly in the Near East, indicate a potential genetic affinity with other oriental breeds renowned for their speed and endurance, such as the Turkmen horse. Highlighting this relationship provides essential context for the observed genetic equilibrium within the Caspian population, potentially reflecting the stability of a historically isolated lineage.

The distribution of genotypes was examined across all breeds, but significant sex-based differences were observed only in Turkmen horses. Specifically, mares show a much higher frequency of the GG genotype (55.5%) compared to stallions (29.7%), whereas stallions exhibit a higher frequency of the TT genotype (29.7% vs. 11.1% in mares).

These differences could be rooted in the breed’s traditional use and the potential selection of traits between males and females. In Turkmen horses, the GG genotype might be associated with traits like strength and robustness, which are crucial for breeding, leading to a higher prevalence in mares. In contrast, the TT genotype might be linked to traits like speed and agility, which are more desirable in stallions used for racing or other competitive purposes. This differentiation could reflect selective breeding practices where stallions are chosen for racing, favoring the T allele. At the same time, mares are selected for their reproductive and nurturing traits, favoring the G allele. Further investigation into the specific effects of these alleles and their association with performance traits could provide a clearer understanding.

## 5. Conclusions

In the present study, we investigated an Acyl-coenzyme A oxidase 1 (*ACOX1*) gene polymorphism, which encodes one of the strategic metabolic enzymes responsible for lipid metabolism. The results confirmed the significantly different genotype and allele distributions of the *ACOX1* gene across horse breeds corresponding to various utility types. Investigation of the native horse breeds in Iran and comparison of them together and with other horse breeds showed a substantial association between the *ACOX1* gene and adaptation to living conditions and the type of physical activity performed. Our study, consistent with previous reports, suggests that ACOX1 could be considered a potential genetic marker for horse performance traits.

## Figures and Tables

**Table 1:** Allele frequency and distribution of genotypes across breeds.

		TT	GG	TG	Number of Horses	G *	T *	HWE
	Arabian	6 (7%)	63 (70%)	21 (23%)	90	0.82	0.18	0.03
	Caspian	2 (10%)	11 (52%)	8 (38%	21	0.71	0.29	0.75
	KWPN	10 (20%)	31 (62%)	9 (18%)	50	0.71	0.29	0.000069
HORSE BREEDS	Kurdish	1 (2%)	63 (86%)	9 (12%)	73	0.92	0.08	0.32
	Thoroughbred	8 (23%)	16 (46%)	11 (31%)	35	0.61	0.39	0.046
	Turkmen	13 (24%)	21 (38%)	21 (38%)	55	0.57	0.43	0.102
	TOTAL	40 (12.34%)	205 (63.27%)	79 (24.38%)	324	0.75	0.25	0.000000

HWE *p*-value; *—allele frequency.

**Table 2:** Table of differences in the distribution of genotypes based on the chi-square test.

	Arabian	Caspian	KWPN	Kurdish	Thoroughbred	Turkmen
Arabian	----					
Caspian	ns	-----				
KWPN	ns	ns	----			
Kurdish	0.0356	0.0028	0.0007	----		
Thoroughbred	0.0117	ns	ns	<0.0001	----	
Turkmen	0.0003	ns	0.0319	<0.0001	ns	----

The numbers in the table are the *p*-values of the comparisons. *p*-value < 0.05 indicates significant differences.

**Table 3:** Distribution of genotypes by breed and sex.

**Genotypes**			**Sex**	
			**Mare Count**	**Stallion Count**
	GG	Arabian	35	28
		Caspian	7	4
		KWPN	14	17
		Kurdish	21	42
		Thoroughbred	4	12
		Turkmen	10 (55.5)	11 (29.7)
	TG	Arabian	12	9
		Caspian	4	4
		KWPN	6	3
		Kurdish	4	5
		Thoroughbred	4	7
		Turkmen	6 (33.3)	15 (40.6)
	TT	Arabian	3	3
		Caspian	1	1
		KWPN	4	6
		Kurdish	0	1
		Thoroughbred	3	5
		Turkmen	2 (11.1)	11 (29.7)

## Data Availability

These data are contained in the article.
